# A cross-sectional investigation of the quality of selected medicines in Cambodia in 2010

**DOI:** 10.1186/2050-6511-15-13

**Published:** 2014-03-05

**Authors:** Naoko Yoshida, Mohiuddin Hussain Khan, Hitomi Tabata, Eav Dararath, Tey Sovannarith, Heng Bun Kiet, Nam Nivanna, Manabu Akazawa, Hirohito Tsuboi, Tsuyoshi Tanimoto, Kazuko Kimura

**Affiliations:** 1 Drug Management and Policy, Faculty of Pharmacy, Institute of Medical, Pharmaceutical and Health Sciences, Kanazawa University, Kakuma-machi, Kanazawa, Ishikawa 920-1192, Japan; 2 Department of Drugs and Food, Ministry of Health, 151-153, Kampuchea Krom St, Khan 7 Makara, Phnom Penh, Cambodia; 3 National Health Product Quality Control Center, Ministry of Health, 151-153, Kampuchea Krom St, Khan 7 Makara, Phnom Penh, Cambodia; 4 Department of Public Health and Epidemiology, Meiji Pharmaceutical University, 2-522-1 Noshio, Kiyose, Tokyo 204-8588, Japan; 5 Faculty of Pharmaceutical Sciences, Doshisha Women’s University, Kodo, Kyotanabe, Kyoto 610-0395, Japan

**Keywords:** Quality of medicine, Spurious/falsely labeled/falsified/counterfeit (SFFC) medicine, Authenticity, Essential medicine, Cambodia

## Abstract

**Background:**

Access to good-quality medicines in many countries is largely hindered by the rampant circulation of spurious/falsely labeled/falsified/counterfeit (SFFC) and substandard medicines. In 2006, the Ministry of Health of Cambodia, in collaboration with Kanazawa University, Japan, initiated a project to combat SFFC medicines.

**Methods:**

To assess the quality of medicines and prevalence of SFFC medicines among selected products, a cross-sectional survey was carried out in Cambodia. Cefixime, omeprazole, co-trimoxazole, clarithromycin, and sildenafil were selected as candidate medicines. These medicines were purchased from private community drug outlets in the capital, Phnom Penh, and Svay Rieng and Kandal provinces through a stratified random sampling scheme in July 2010.

**Results:**

In total, 325 medicine samples were collected from 111 drug outlets. Non-licensed outlets were more commonly encountered in rural than in urban areas (*p* < 0.01). Of all the samples, 93.5% were registered and 80% were foreign products. Samples without registration numbers were found more frequently among foreign-manufactured products than in domestic ones (*p* < 0.01). According to pharmacopeial analytical results, 14.5%, 4.6%, and 24.6% of the samples were unacceptable in quantity, content uniformity, and dissolution test, respectively. All the ultimately unacceptable samples in the content uniformity tests were of foreign origin. Following authenticity investigations conducted with the respective manufacturers and medicine regulatory authorities, an unregistered product of cefixime collected from a pharmacy was confirmed as an SFFC medicine. However, the sample was acceptable in quantity, content uniformity, and dissolution test.

**Conclusions:**

The results of this survey indicate that medicine counterfeiting is not limited to essential medicines in Cambodia: newer-generation medicines are also targeted. Concerted efforts by both domestic and foreign manufacturers, wholesalers, retailers, and regulatory authorities should help improve the quality of medicines.

## Background

Spurious/falsely labeled/falsified/counterfeit (SFFC) medicines are deliberately and fraudulently mislabeled with respect to identity or source [1,2]. Falsifying is greatest in those regions where regulatory and legal oversight is weakest. Although precise, detailed data on SFFC medicines are difficult to obtain, estimates range from less than 1% of sales in developed countries to over 10% in developing countries, depending on the geographic area [3]. Several reports have documented adverse health consequences from consumption of SFFC medicines. More than 200 children died in a hospital in Bangladesh through ingesting SFFC paracetamol, which contained diethylene glycol, in 1990–93 [4]. In 1998, 30 infants died from SFFC paracetamol in India [5]. In Southeast Asia in 2001, 38% of 104 anti-malaria drugs were found to be SFFC [6]. In Cambodia in 1999, at least 30 people died through SFFC artesunate containing sulfadoxine-pyrimethamine, which is an older, less effective anti-malarial [5]. In 2005, a man aged 23 years treated with SFFC artesunate died in eastern Burma (Myanmar) [7]. In 1999, two people died through taking fake anti-diabetic medicines containing illegal quantities of glibenclamide in China; in 2008, four people died in Singapore after taking an SFFC phosphodiesterase type 5 inhibitor containing glibenclamide [8,9]. In addition to SFFC products, substandard and degraded medicines are classified as poor-quality medicines [10]. Substandard medicines are genuine medicines produced by legitimate manufacturers that do not meet the quality specifications declared by the producer [11]. Degraded products may result from exposure of good-quality medicines to light, heat, and humidity; however, it can be difficult to distinguish degraded medicines from those that left the factory as substandard [12]. Substandard and degraded medicines reduce the effectiveness of therapy.

In Cambodia, the Ministry of Health (MoH) reported in 2001 that 13% of medicines were SFFC, with 21% being substandard and 50% unregistered [13]; in the same country, 35 medicines were found to be SFFC among 142 products in 2004 [14]. Furthermore, in our previous study in Cambodia in 2006–09, we identified 19 SFFC products among life-saving essential medicines [15,16]. Evidence suggests that lack of awareness and inappropriate management in the supply chain could facilitate the distribution of SFFC medicines in the country [17].

To oppose the threat of SFFC medicines and prevent their spread, the Department of Drugs and Food (DDF) of the MoH has been conducting surveillance on the quality of medicines since 2006. In 2010, we selected some newer-generation medicines, including antibiotics, to assess falsifying trends in the context of this developing country. At the same time, we sought to identify the influential factors in falsifying practices and develop a cost-effective surveillance system to ensure the quality of medicines.

## Methods

### Sampling area

Samples were collected from the capital, Phnom Penh (urban area), and Svay Rieng and Kandal provinces (rural areas). The list of licensed outlets in Phnom Penh was obtained from the DDF, MoH of Cambodia, in June 2013. The calculated sample size was 193 pharmacies, 58 depot A outlets, and 79 depot B outlets, with an alpha value of 5 and power of 0.8. In Phnom Penh, as many private drug outlets as possible of the four types of private drug outlets (i.e., pharmacies, depot A, depot B, and non-licensed outlets) were visited within the period of sample collection. For sampling purposes, a stratified random scheme developed by means of random number tables was used. All the outlets found in Svay Rieng and all illegal outlets found in the sampling area were visited. Additionally, some samples were collected from wholesalers and drug outlets in Kandal province while the sampling teams moved between Phnom Penh and Svay Rieng. In Cambodia, a pharmacy outlet is run by a registered pharmacist, a depot A outlet by an assistant pharmacist (with 4 years’ pharmacy training), and a depot B outlet by a retired midwife or nurse [17]. The sampling areas were selected in consultation with the MoH, taking into account the degree of urbanization, population density, concentration of drug outlets, budgetary limitations, and geographic importance in sharing a border with another country.

### Sample collection

Cefixime tablets, omeprazole capsules, co-trimoxazole (a combination of sulfamethoxazole and trimethoprim) tablets, clarithromycin tablets, and sildenafil tablets were selected as candidate medicines in consultation with the DDF. Cefixime, omeprazole, clarithromycin, and sildenafil were chosen in 2010 as newer-generation medicines; this was in contrast to selection from a list of essential medicines in our previous studies [15,16]. Sample information was collected using a sampling form that included the contents of the packages sold, price, and outlet information. Samples were collected between June 7 and 15, 2010 by two teams. Each team consisted of a research investigator, a locally recruited sampling officer, and a sampling assistant. The locally recruited members were provided with training before sampling and instructed to purchase medicines. The sampling officer purchased medicines in an outlet and completed a sampling form for each sample. Medicines collected from the same outlet and labeled with the same international non-proprietary name, brand name, strength, size, batch/lot number, and manufacturing and expiry dates were considered one sample. For authentication purposes, the teams collected containers or packages for most of the samples. Samples were preserved at 20–25°C until analysis.

### Observation test

Details of the packaging condition and the label information of the samples were carefully noted. Compliance with the Association of Southeast Asian Nations Common Technical Dossier (ACTD) for the registration of pharmaceuticals for human use (to which drug registration in Cambodia conforms) was examined [18]. Bar codes were also recorded.

### Materials for quality evaluation

United States Pharmacopeia (USP) reference standards of omeprazole, sulfamethoxazole, trimethoprim, and clarithromycin-related compound A (6,11-di-*o*-methyl erythromycin A) were purchased from the Reference Standard Center, Bureau of Drugs and Narcotics, Thailand (Nonthaburi, Thailand). Reference standards of cefixime, clarithromycin, and sildenafil citrate were generously donated by Astellas Pharma Inc. (Tokyo, Japan), Taisho Toyama Pharmaceutical Co, Ltd. (Tokyo, Japan), and Pfizer Japan Inc. (Tokyo, Japan), respectively. Metronidazole and butyl *p*-hydroxy benzoate were purchased from Nacalai Tesque, Inc. (Kyoto, Japan). Primidone and sulfadoxine were purchased from Wako Pure Chemical Industries, Ltd. (Osaka, Japan). Lansoprazole was purchased from Sigma-Aldrich Co. LLC (St. Louis, MO, USA). Methanol and acetonitrile of high-performance liquid chromatography (HPLC) grade was purchased from Nacalai Tesque, Inc. (Kyoto, Japan). All other chemicals were commercially available and of analytical grade.

### Quality evaluation

The Medicine Quality Assessment Reporting Guidelines (MEDQUARG) were followed when reporting in generally [10].

To assess the pharmaceutical quality of the samples, active ingredients of the samples were quantified by HPLC using ultraviolet detection (Shimadzu, Kyoto, Japan). The system suitability for analysis of each medicine was verified according to USP 30. A linear relationship between the peak area and concentration of each reference standard was observed within the range of 25–200% of the active ingredient (r^2^ = 0.999–1.000), and the assay was performed within that range. The intra- and inter-day coefficient of variation was less than 3.0%. In addition, the methods were validated as being repeatable and accurate (n = 6). Metronidazole, butyl *p*-hydroxybenzoate, primidone, lansoprazole, and sulfadoxine were used as internal standards in the analysis of cefixime, clarithromycin, co-trimoxazole, omeprazole, and sildenafil, respectively. The samples were analyzed between July 2010 and March 2012 in Kanazawa University. The quality evaluation was completed within the expiry date for each sample.

For cefixime tablets, omeprazole capsules, co-trimoxazole tablets, and clarithromycin tablets, the assay, a content uniformity test, and dissolution test were performed with reference to USP 30, USP34, or British Pharmacopeia (BP) 2010 as indicated on the package insert or outer package.

For sildenafil tablets, the assay was performed according to the method described previously [19]. In each sample, three or six tablets were analyzed. The acceptable range was set as follows: in the quantity test, sildenafil tablets containing not less than 90% and not more than 110% on an average quantity taken from 10 units, and no unit less than 75% or more than 125% of the labeled amount of sildenafil; in the dissolution test, the acceptable range was an average dissolution rate in 3 or 6 units equal to or greater than 75% and no unit less than 50%, with a dissolution time of 15 minutes. The content uniformity test could not be conducted because of insufficient material.

### Authenticity investigation

The methodology of the authenticity investigation and registration verification was adopted from the World Health Organization [15,16,20,21]. Label information on the packages and containers was cross-checked with a database prepared after collection. Photographs of each sample, its packaging, and package inserts were obtained for such purposes. These data were then catalogued. A database of manufacturer addresses was also prepared using printed information, Web searches, and e-mail and telephone communication. Portions of all samples were then sent to the respective manufacturers, requesting verification of their products. Information on the manufacturers and their medicines was requested from medicine regulatory authorities of the countries in which the manufacturing took place. Furthermore, each sample’s registration was confirmed by the DDF.

### Statistical analysis

Considering the limitations of the small sample size, descriptive analysis was performed using SPSS 19.0.0 (IBM SPSS Inc, Chicago, IL, USA). Where appropriate, Fisher’s exact test was used to test the significance of categorical variables. Statistical significance was evaluated at the 5% level.

## Results

Table 1 presents an outline of the samples. We collected a total of 325 samples from 111 drug outlets (including 14 wholesalers) in the study area. Of these, 60 (18.5%) were cefixime, 48 (14.8%) were clarithromycin, 91 (28.0%) were omeprazole, 44 (13.5%) were sildenafil, and 82 (25.2%) were co-trimoxazole. Of the samples, 237 (72.9%) were collected from Phnom Penh and 88 (27.1%) from the provinces: 81 (24.9%) from Svay Rieng and seven (2.2%) from Kandal. Foreign products constituted the majority (80%) of the total samples. Local manufacture was commonest for co-trimoxazole (48/82 samples).

**Table 1:** Outline of the samples

	**Sampling area**		**Shop category**					**Country of manufacturer**	
	**Urban**	**Rural**	**Pharmacy**	**Depot A**	**Depot B**	**Wholesaler**	**Non-licensed outlet**	**Domestic**	**Imported**
Cefixime (n = 60)	42	18	20	10	19	6	5	4	56
Clarithromycin (n = 48)	36	12	17	10	15	4	2	10	38
Co-trimoxazole (n = 82)	58	24	21	16	28	7	10	48	34
Omeprazole (n = 91)	63	28	26	21	24	8	12	2	89
Sildenafil (n = 44)	38	6	24	5	6	7	2	0	44
Total (n = 325)	237	88	108	62	92	32	31	64	261

### Drug outlets

Of the 325 samples, we collected 108 (33.2%) from pharmacies, 62 (19.1%) from depot A outlets, 92 (28.3%) from depot B outlets, 32 (9.8%) from wholesalers, and 31 (9.5%) from non-licensed outlets. We found non-licensed outlets significantly more frequently in rural (33.0%, 29/88) than in urban areas (0.8%, 2/237) (Fisher’s exact test: *p* < 0.01). Pharmacists were present in 72.4% (21/29) of the pharmacies. All 14 wholesalers but only one pharmacy had air conditioning.

### Observations

We observed differences in package design (layout and/or printed colors) in four products—one each of cefixime and clarithromycin and two kinds of omeprazole—from the same lot number. With eight products—two kinds of cefixime, three kinds of clarithromycin, one co-trimoxazole, and two kinds of omeprazole—we observed a different package design with different lot numbers. Except for nine samples, ACTD requirements (which were fully implemented in early 2011) appeared in the information on the packaging. Six of those samples lacked lot numbers and manufacturing and expiry dates on their packaging. With three of those samples, the packaging did not include a manufacturing date. The registration number did not appear on the outer packaging with some samples (Table 2). One sample of cefixime bore a false lot number, had two spelling errors, and was confirmed as an SFFC medicine in the authenticity investigation.

**Table 2:** Information on outer packaging

**Items required by ACTD**	**Presence**		**Absence**	
	**n**	**(%)**	**n**	**(%)**
Batch number	313	(98.1)	6	(1.9)
Manufacturing date	310	(97.2)	9	(2.8)
Expiration date	313	(98.1)	6	(1.9)
Registration number	304	(95.3)	15	(4.7)

n = 319 samples.

About 80% (264/325) of the samples included a European Article Number code, which is a bar-code symbol used in supply chain management. However, 61 products had neither a European Article Number code nor a GS1 DataBar™ symbol (GS1, Brussels, Belgium), which can carry more information and be used to identify small items.

We observed varying degrees of discrepancies in compliance with ACTD in the information that appeared in package inserts (Tables 3 and 4). We collected package inserts from 106 products (284 samples, 87.4%). The package inserts of 102 products (87.2%) were written in English and/or Khmer and/or French: all these languages are officially accepted in Cambodia. However, four products were written in Vietnamese only (Table 3). Table 4 shows other discrepancies with the package inserts of 87 products, which were written in English. The indication, dosage, and administration were described for all products (Table 4). One package insert failed to stipulate contraindications, two did not state precautions, and two did not indicate side effects; one product lacked all three of these items (Table 4). With over 10% of the products, information relating to clinical pharmacology (23 products), drug interactions (11 products), pregnancy and lactation (12 products), overdose and treatment (33 products), and storage conditions (20 products) was not provided (Table 4). The date of revision of the package insert was not given for 79 products (90.8%). Six (6.9%) products satisfied the items required by ACTD, whereas 55 (63.2%) products had missing data for multiple elements.

**Table 3:** Language of package inserts

**Language**	**n**	**(%)**
Khmer only	1	(0.9)
English only	74	(63.2)
English and Khmer	5	(4.3)
Khmer and other language	11	(9.4)
English and other language	8	(6.8)
French only	3	(2.6)
Vietnamese only	4	(3.4)
Unavailable	11	(9.4)
Total	117	(100.0)

**Table 4:** Information on package inserts

**Items required by ACTD**	**Presence**		**Absence**	
	**n**	**(%)**	**n**	**(%)**
Clinical pharmacology	64	(73.6)	23	(26.4)
Indications	87	(100.0)	0	(0.0)
Dosage and administration	87	(100.0)	0	(0.0)
Contraindications	86	(98.9)	1	(1.1)
Warnings and precautions	85	(97.7)	2	(2.3)
Drug interactions	76	(87.4)	11	(12.6)
Pregnancy and lactation	75	(86.2)	12	(13.8)
Side effects	85	(97.7)	2	(2.3)
Overdose and treatment	54	(62.1)	33	(37.9)
Storage conditions	67	(77.0)	20	(23.0)
Date of revision	8	(9.2)	79	(90.8)

n = 87 products, whose package inserts were written in English.

We collected products that were unregistered or lacked lot numbers, manufacturing dates, expiry dates, or registration numbers (in at least one item) from illegal outlets (7/24) more frequently than from legal ones (15/279) (Fisher’s exact test: *p* < 0.05). However, the sampling area and origin of the samples were not statistically associated as acceptable findings.

### Pharmaceutical quality

Among the 325 samples analyzed for their contents, 47 (14.5%) were of unacceptable quality (Table 5). Of 281 samples, we finally determined that 15 (4.6%) were unacceptable in content uniformity tests. In dissolution tests, which were not obligatory for registration in 2010 in Cambodia, 80 (24.6%) samples were unacceptable. Interestingly, all the finally unacceptable samples (15 of 281 samples) in the content uniformity tests were of foreign origin and were registered products. Of the 64 domestic samples, 21 were unacceptable for one or more of the quality tests.

**Table 5:** Quality test results

	**Number of collected samples**	**Quantity test**			**Content uniformity test**			**Dissolution test**		
		**Accepted**	**Unaccepted**	**Pending**	**Accepted**	**Unaccepted**	**Pending**	**Accepted**	**Unaccepted**	**Pending**
Cefixime	60	56	3	1	55	0	5	51	3	6
Clarithromycin	48	44	4	0	42	1	5	27	11	10
Co-trimoxazole	82	73	9	0	81	0	1	65	15	2
Omeprazole	91	54	22	15	31	14	46	42	45	4
Sildenafil	44	32	9	3	-	-	-	28	6	10
Total	325	259	47	19	209	15	57	213	80	32

Pending: samples pending at time of decision because of insufficient material.

### Authenticity

**Figure 1:**
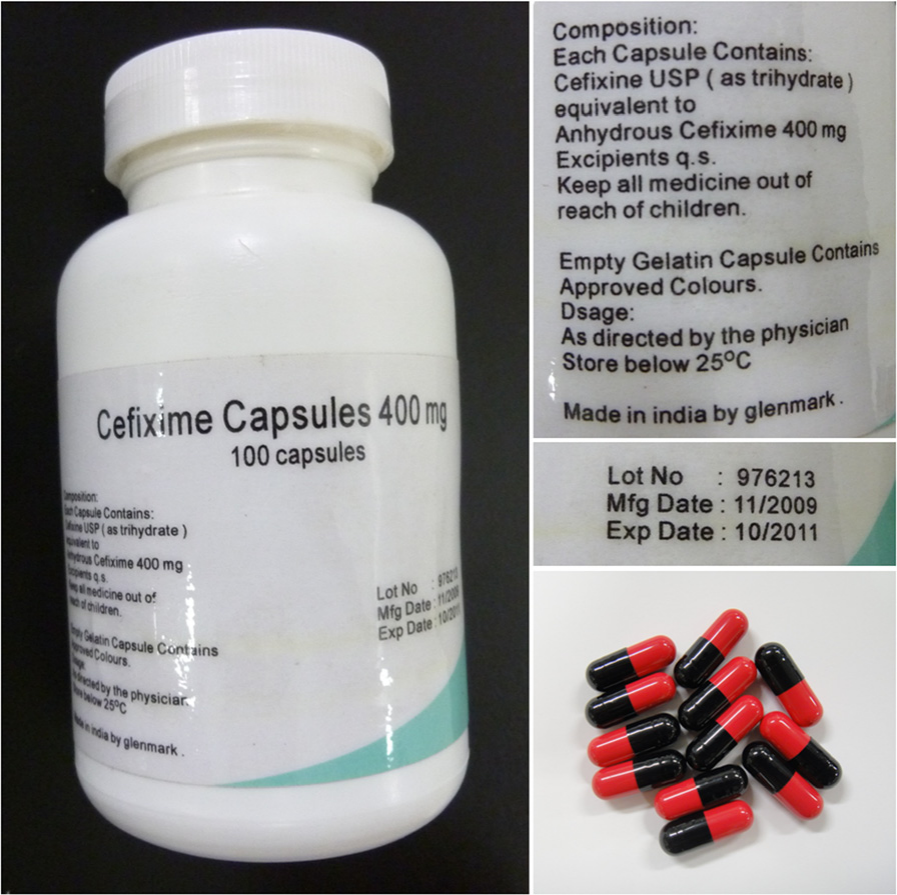
Bottle and label for SFFC cefixime sample.

We received authentication reports from 49 of 75 (65.3%) manufacturers for 230 (71%) samples and 5 of 11 (45.5%) medicine regulatory authorities. On the basis of authenticity results, we confirmed one sample of cefixime as an SFFC medicine (Figure 1). The sample product was not registered in Cambodia; it was labeled with batch number 976213, manufactured date 11/2009, and expiry date 10/2011. The product was purchased from a pharmacy, and it came from an unknown wholesaler. The sample passed quantity, content uniformity, and dissolution test.

Of the 325 samples, 309 (95.1%) were registered by the DDF and 16 (4.9%) were not registered. Six samples of unregistered medicines were sold with labels indicating that they were registered. Two of these samples were supplied for use in the public sector (hospitals or health centers) only; however, they were purchased from pharmacies. All 16 samples of such illegal medicines were imported products.

## Discussion

The results of this survey indicate a number of problems in the manufacturing and distribution conditions, quality, and packaging of medicines in Cambodia. Among 111 drug outlets we visited, including wholesalers, only one pharmacy in Phnom Penh and all 14 wholesalers had air conditioning in their storage areas. Despite temperature rises above 40°C in the dry season in Cambodia, the other drug outlets did not have any temperature-control measures [22]. Storing medicines at high temperature and humidity could result in deterioration of quality and might facilitate the distribution of degraded medicines in the market [23-25]. In Cambodia, good pharmacy practice, distribution practice, and storage practice have not been implemented. Hence, the deterioration in quality may be due to storage or distribution. To prevent the distribution of degraded products, storage conditions at drug outlets need to be better controlled. To this end, good pharmacy practice will be introduced in Cambodia by the end of 2013.

Following quality evaluation of the collected samples, we found that some products had problems in their outer packaging, such as different colors and/or layout for the same product, spelling errors, and a lack of identification codes. These discrepancies may have resulted from insufficient awareness of the risks and countermeasures adopted by legitimate manufacturers against SFFC or substandard medicines. Such shortcomings could make it more difficult to distinguish SFFC medicines, and dealing with this problem should help prevent the entry of such products into the market. Additionally, the circulation of unregistered medicines and unauthorized distribution of publicly donated ones could affect the emergence of SFFC medicines in the supply chain. The SFFC medicine we detected in this survey—cefixime tablets bearing a false lot number—did not have Cambodian registration, bore spelling errors on the label, and was obtained at a pharmacy. Nevertheless, we found the sample to be acceptable in quantity, content uniformity, and dissolution tests according to USP 30. Cefixime, an oral third-generation cephalosporin, has excellent therapeutic action against infections caused by bacteria and is relatively high in price. Counterfeiters thus have an incentive in producing such antibiotics [26]. This is possibly the first report of SFFC cefixime in Cambodia. The findings of this survey indicate that not only are earlier-generation, commonly used essential medicines targeted by counterfeiters, newer-generation medicines are similarly falsified because of a better profit margin. Sildenafil, a newer-generation medicine, is a medicine that is more likely to be counterfeited in developed countries [27]. The present study shows that sildenafil had low distribution, and no counterfeit product was found. This finding suggests that the target medicine for counterfeiting may vary depending on the country.

In the pharmaceutical quality evaluation of the 325 samples we identified, 103 (31.7%) were unacceptable in one or more of the quantity, content uniformity, and dissolution tests. Most of those were enteric-coated omeprazole capsules, which especially showed an unacceptably high failure ratio in the dissolution test. Unacceptable samples in the dissolution test for omeprazole may have resulted from poor enteric coating, which could cause degradation of the omeprazole by gastric acid and lead to ineffectiveness after oral administration. Variables related to formulation or manufacturing processes of medicines can significantly affect dissolution and the expected medical effects. Consequently, some pharmaceutical manufacturers, mainly from overseas, may not have attained the necessary technical level to produce enteric preparations. To maintain the quality of medicines, exporting countries should not allow substandard medicines to be exported to other developing countries. Furthermore, each manufacturer selects its specifications among existing pharmacopoeias and in-house specifications; thus, for some medicines, the conditions in dissolution testing are different between USP and BP standards. Indeed, it would be desirable for the MoH to define uniform requirements for dissolution testing. In early 2011, the MoH began implementing the dissolution test for registration; there was, however, no focus on this issue before 2011. Thus, samples collected in 2010 may have been particularly subject to problems with the dissolution test. Since dissolution testing has been added as a mandatory requirement for registering medicines in Cambodia, the situation may improve in the near future. Furthermore, the lack of information in package inserts and non-compliance with linguistic requirements of the ACTD could hinder the safe, proper use of medicines. Having adequate information on the packaging and inserts could minimize the misuse of medicines and reduce therapeutic failure [28,29]. For package inserts, three languages—French, English, and Khmer—are officially accepted in Cambodia. To encourage the proper use of medicines, some key words have been translated into Khmer by the MoH for products lacking Khmer instructions. Essentially, the contents of all package inserts need to be translated into Khmer from French or English. Furthermore, ACTD was implemented in Cambodia only at the end of 2010. Before 2008, there was no checking and verification of all registered products by design and brand name. Therefore, the MoH has carried out such checking and verification only since 2008. However, products registered before 2008 can be distributed and sold as usual until their licenses expires; it is not until the papers relating to such products are submitted for license renewal that the ACTD requirements come into effect. ACTD was fully implemented in early 2011.

Printing technology has improved and become widely available to the point where SFFC medicines are able to show very close similarity to original products [21,30]. Even medical practitioners may fail to spot SFFC medicines in their practices. Stopping SFFC medicines from entering the market is one way of preventing consumers being subjected to the health hazards associated with such medicines.

Toward creating a healthy, secure system for pharmaceutical distribution, it is likewise imperative to stop SFFC medicines from entering the market. Improved regulation of illegal medicines, revision and compliance of existing rules for pharmaceuticals, and introducing a pharmaceutical traceability system could help ensure the circulation of genuine medicines.

The collected samples in this study may not have been representative of conditions throughout Cambodia because of the limited area of sample collection, insufficient sample size in the stratified random sampling in Phnom Penh, and convenient sampling of some samples from selected drug outlets. Additionally, we did not receive responses relating to the authenticity investigation from 26 manufacturers; therefore, we were unable to confirm 95 samples as having been genuine or SFFC. Because of the insufficient number of samples, we could not verify the status of 43 medicines in our quality evaluation. These limitations make it difficult to assess the actual extent of SFFC and substandard medicines in the entire Cambodian supply chain.

## Conclusions

In conclusion, the results of this survey indicate that medicine falsifying is not limited to essential medicines in Cambodia: newer-generation medicines are also targeted. Concerted efforts by manufacturers—both domestic and overseas—along with those by wholesalers, retailers, and regulatory authorities are necessary to ensure the quality of medicines.

## Abbreviations

MoH: the Ministry of Health; DDF: the Department of Drugs and Food; ACTD: the Association of Southeast Asian Nations Common Technical Dossier; USP: United States Pharmacopeia; HPLC: high-performance liquid chromatography; BP: British Pharmacopeia.

## Competing interests

The authors declare that they have no competing interests.

## Authors’ contributions

NY, HTa, HBK, NN, MA, and KK conceived and designed the experiments. NY, HTa, ED, TS, TT, and KK performed the experiments. NY, MHK, and HTa analyzed the data. TT and KK contributed reagents/materials/analysis tools. NY and MHK wrote the first draft of the manuscript. ED, HBK, NN, MA, HTs, TT, and KK contributed to the writing of the manuscript. All authors read and approved the final manuscript.

## Pre-publication history

The pre-publication history for this paper can be accessed here:

http://www.biomedcentral.com/2050-6511/15/13/prepub
